# Chronic *vs.* Short-Term Acute O_3_ Exposure Effects on Nocturnal Transpiration in Two Californian Oaks

**DOI:** 10.1100/tsw.2007.33

**Published:** 2007-03-21

**Authors:** N. E. Grulke, E. Paoletti, R. L. Heath

**Affiliations:** ^1^ PSW Research, Station USDA Forest Service, Riverside, CA, USA; ^2^ IPP-CNR, Florence, Italy; ^3^ Botany and Plant Sciences Department, University of California, Riverside, CA, USA

**Keywords:** ozone exposure, nighttime transpiration, Quercus kelloggii, Quercus douglasii

## Abstract

We tested the effect of daytime chronic moderate ozone (O_3_) exposure, short-term acute exposure, and both chronic and acute O_3_ exposure combined on nocturnal transpiration in California black oak and blue oak seedlings. Chronic O_3_ exposure (70 ppb for 8 h/day) was implemented in open-top chambers for either 1 month (California black oak) or 2 months (blue oak). Acute O_3_ exposure (~1 h in duration during the day, 120–220 ppb) was implemented in a novel gas exchange system that supplied and maintained known O_3_ concentrations to a leaf cuvette. When exposed to chronic daytime O_3_ exposure, both oaks exhibited increased nocturnal transpiration (without concurrent O_3_ exposure) relative to unexposed control leaves (1.8× and 1.6×, black and blue oak, respectively). Short-term acute and chronic O_3_ exposure did not further increase nocturnal transpiration in either species. In blue oak previously unexposed to O_3_, short-term acute O_3_ exposure significantly enhanced nocturnal transpiration (2.0×) relative to leaves unexposed to O_3_. California black oak was unresponsive to (only) short-term acute O_3_ exposure. Daytime chronic and/or acute O_3_ exposures can increase foliar water loss at night in deciduous oak seedlings.

## INTRODUCTION

The occurrence of nocturnal transpiration has been cited as an adaptation in both nutrient- and water-limited environments. Nocturnal transpiration under drought conditions helps to redistribute water from hydrologically deep sources to near-surface roots. Root water loss in near-surface soil horizons increases nutrient availability and potential for uptake in soil immediately adjacent to fine roots[1]. Nocturnal transpiration has also been found in plants drought stressed the previous day. In this case, nocturnal transpiration is proportional to the level of daytime drought stress and is thought to promote rehydration of foliage[2].

Nocturnal transpiration has also been reported previously for three species experimentally exposed to moderate daytime ozone ($O_3$) exposures[3,4]. In these studies, nighttime transpirational losses were 12–

50% that of daytime transpirational rates. In a fourth study, moderately high to high exposure of both $O_3$ and $NO_x$ was correlated to nocturnal transpiration averaging 11% that of daytime values[5]. In these cases, loss of stomatal control at night is likely to be the result of mechanical failure of the guard cells in the presence of strong oxides. Ozone exposure is known to modify cell membrane permeability to $K^+$[6], to modify $Ca^{++}$-based signaling[7], and to alter the chemical structure of cell walls such that water loss is buffered and slows guard cell response to changing conditions of water potential (e.g., delignification)[8].

In this paper, we report the effects of daytime $O_3$ exposure on nocturnal transpiration in seedlings of two deciduous trees: California black oak and blue oak. Chronic medium-term $O_3$ exposure, acute short-term $O_3$ exposure, and the combined effects of both were employed to test effects on nocturnal transpiration relative to leaves unexposed to $O_3$.

## MATERIALS AND METHODS

### Plant Material

Two broadleaf, deciduous oak species were chosen for flux measurements: California black oak (*Quercus kelloggii* Newb.) and blue oak (*Q. douglasii* Hook & Arn). Although botanical references state that blue oak is deciduous, blue oak retains up to 3 years of leaves in the foothills of central and the southern Sierra Nevada (N. Grulke, field observations). Blue oak seedlings in this experiment retained 3 years of leaves, but response of only current-year leaves was measured. Oaks were germinated from acorns and grown in greenhouses until 3–5 years old. There was no evidence that these seedlings were hybrids with any other oak species.

### Experimental Design

In mid-March 2005, seedlings were placed in open-top exposure chambers and subjected to a chronic $O_3$ exposure of 70 ppb for 8 h/day for 1 (California black oak) or 2 months (blue oak). One or two plants of each oak species were placed in three charcoal-filtered and three elevated open-top chambers. Oaks exposed to chronic $O_3$ exposure or to activated charcoal-filtered air were transferred to the greenhouse in the early morning of experimental acute $O_3$ exposure. The maximum $O_3$ concentration observed in the greenhouse was 10 ppb. Adjacent leaves of oaks on the same plant were exposed to either no $O_3$ exposure (using a commercial gas exchange system) or ~1 h of known $O_3$ exposure (120–220 ppb) using a novel gas exchange system[9] briefly described below. The whole plant was exposed to daylight within the greenhouse, and leaves in the cuvettes were exposed to constant light (850 $\mu$mol $m^{-2}$ $sec^{-1}$, photon flux density), ambient temperature (22–28ºC), and relative humidity (22–60%). The following night, gas exchange of the paired leaves (with and without prior $O_3$ exposure) was measured with a carefully calibrated, open gas exchange system (Model 6400, LiCor Instruments, Lincoln, NE) with no exposure to light at 390 ppm $CO_2$. During gas exchange measurements at night, ambient conditions in the cuvette were matched with that of the greenhouse: leaf VPD averaged 1.3 kPa, relative humidity averaged 34%, and leaf temperature averaged 18ºC.

### $O_3$ Exposure Gas Exchange System

We designed a novel gas exchange system to test the effects of $O_3$ exposure alone on foliar gas exchange[9] and to measure $O_3$ flux to leaves directly. We were unable to use commercial gas exchange systems for this purposed because 95% of the $O_3$ supplied to commercial cuvettes is adsorbed by the internal surfaces. In brief, the source of the air flow was premixed $CO_2$ (in air), humidified and cooled prior to the cuvette. A low flow of high concentration of $O_3$ was metered into the cooled, humidified air stream with a mass flow controller. Matched custom $O_3$ monitors, designed for both low flow (120 sccm) and fast response (20 sec), were used to measure $O_3$ concentration, one before (reference) and one after (sample) the cuvette. After $O_3$ measurements, air was passed to either the reference or sample $CO_2$ and $H_2O$ infrared gas analyzers, respectively, then exhausted. Ozone concentration was measured in an empty cuvette with stable temperature and humidity, before and after leaf gas exchange measurements. Although covered with Teflon film, cuvette surfaces adsorbed 10–30% of $O_3$ delivered, and null cuvette $O_3$ adsorption was accounted for in all measurements of foliar $O_3$ uptake. After $O_3$ flux was measured in the null cuvette, one leaf on a plant was inserted into the $O_3$-exposure cuvette, and an adjacent leaf on the same plant was simultaneously measured using an $O_3$-free commercially available LiCor (model 6400) gas exchange system. Light level, leaf temperature, air flow, and cuvette fan speed were matched in the two systems.

## RESULTS

### California Black Oak

One month of chronic $O_3$ exposure during the daytime increased foliar nocturnal transpiration (without concurrent $O_3$ exposure or exposure to light) by 1.8× relative to foliage not exposed to $O_3$ (Fig. 1). Nocturnal transpiration resulting from chronic $O_3$ exposure was 30% of daytime values (based on a maximum daytime value observed of ~1.8 mmol $m^{-2}$ $sec^{-1}$). Although there was an increase in nocturnal transpiration in response to short-term acute $O_3$ exposure, differences were not statistically significant.

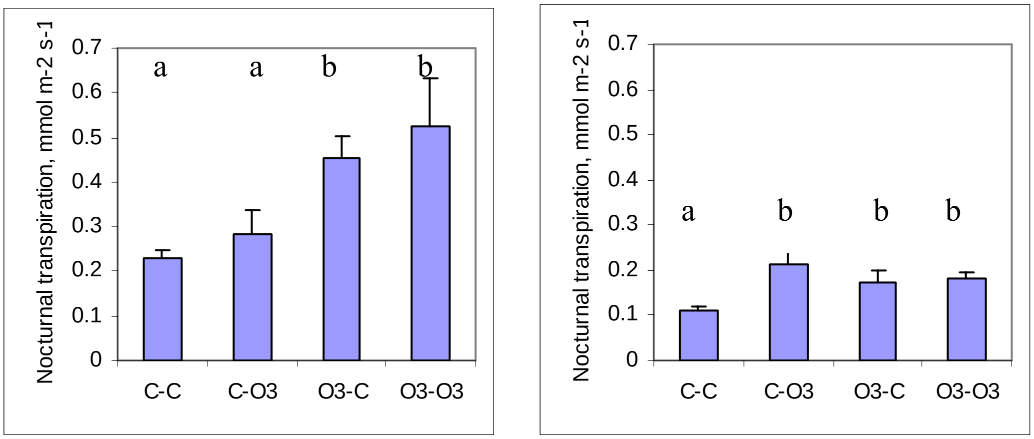

**FIGURE 1.** Nocturnal transpiration of California black oak leaves (on left) and blue oak leaves (on right) measured after daytime exposure, with no ambient light or pre-exposure to light. **C-C**: plants exposed to activated charcoal-filtered (CFA) air with no short-term exposure to $O_3$; **C-$O_3$**: plants exposed to CFA with short-term acute $O_3$ exposure; **$O_3$-C**: plants exposed to chronic $O_3$ exposure with no short-term exposure to $O_3$; **$O_3$-$O_3$**: plants exposed to both chronic and acute $O_3$ exposure. Error bars indicate $\pm$ 1 S.E. for four plants in each group. Different letters indicate statistical differences at the $p = 0.05$ level between bars.

To display the relationship between nocturnal transpiration (T) and $O_3$ dose, we plotted the ratio of T with short-term acute $O_3$ to T without $O_3$ vs. $O_3$ dose experienced in the short-term acute $O_3$ exposures (Fig. 2, left). Because the numerator and denominator represent gas exchange of different leaves (at night), and the duration of exposure and $O_3$ concentration varied with each short-term exposure, there was significant variability in response in the graphed response. For California black oak, $O_3$ doses $<\sim 200$ ppb h had a relatively small effect on nocturnal transpiration and the regression line was not statistically significant.

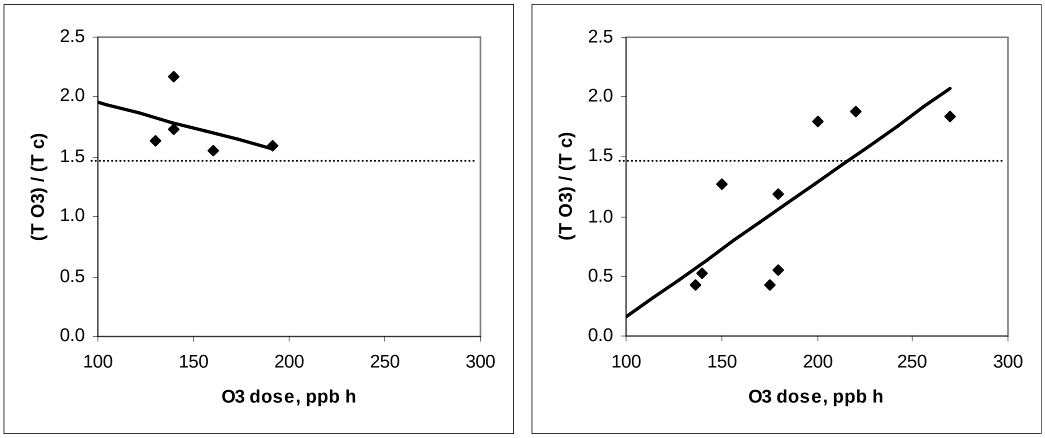

**FIGURE 2.** The ratio of nocturnal transpiration (T) with acute $O_3$ exposure divided by the transpiration of an adjacent leaf with no $O_3$ exposure, plotted as a function of the daytime short-term acute $O_3$ exposure. The dashed line indicates a 1:1 ratio of T which would be expected if there were no effect of short-term, acute $O_3$ exposure. There was no statistically significant regression fit for California black oak (left). A linear regression was significant for blue oak (right).

## Blue Oak

Two months of chronic $O_3$ exposure during the daytime increased foliar nocturnal transpiration (without concurrent $O_3$ exposure or exposure to light) by 1.6× relative to foliage not exposed to $O_3$ (Fig. 1). Nocturnal transpiration resulting from this chronic $O_3$ exposure was 16% of daytime values (based on a maximum daytime value observed of ~1.5 mmol $m^{-2}$ $sec^{-1}$). For plants with no prior $O_3$ exposure (controls grown in activated charcoal-filtered, open-top chambers), short-term (1 h) acute $O_3$ exposure significantly increased nocturnal transpiration 2.0× that of control leaves. Nocturnal transpiration resulting from this acute $O_3$ exposure was 24% of daytime values. There were no synergistic effects of both chronic moderately high and short-term acute $O_3$ exposure.

The ratio of T with short-term acute $O_3$ to T without $O_3$ was plotted against $O_3$ dose experienced in the short-term acute $O_3$ exposures (Fig. 2, right). The ratio increased with increasing $O_3$ dose and the regression was statistically significant ($p < 0.05$). Ozone doses exceeding 180 ppb h significantly increased nocturnal transpiration in leaves exposed to short-term acute $O_3$ exposure.

The response of both deciduous oaks was combined to illustrate the effect of both chronic and acute $O_3$ exposure on nocturnal transpiration (Fig. 3). The lower line represents the transpirational response to short-term acute exposure for leaves with no prior $O_3$ exposure (plotted vs. dose). The upper line represents the transpirational response to short-term acute exposure for leaves chronically exposed to $O_3$. In both datasets, the values on the y-axis (representing 0 dose) are transpiration of the control leaves in each treatment (CFA or chronic $O_3$ exposure, respectively) without short-term acute $O_3$ exposure. The lower regression line is a statistically significant fit ($p < 0.05$), but there were no differences in y-intercept or slope between the two lines. The response lines suggest that (1) short-term acute $O_3$ exposure increases nocturnal transpiration, (2) chronic $O_3$ exposure increases overall nocturnal transpiration, and (3) the interaction between chronic and acute $O_3$ exposures was not synergistic.

## DISCUSSION

Stomates may remain open at night and there is an apparent net efflux of water from leaves, after daytime long-term chronic or short-term acute $O_3$ exposure. Foliage of California black oak and blue oak seedlings exhibited elevated nocturnal transpiration when exposed to chronic daytime $O_3$ exposure. Short-term acute $O_3$ exposure during the day did not significantly (additionally) enhance nocturnal transpiration in

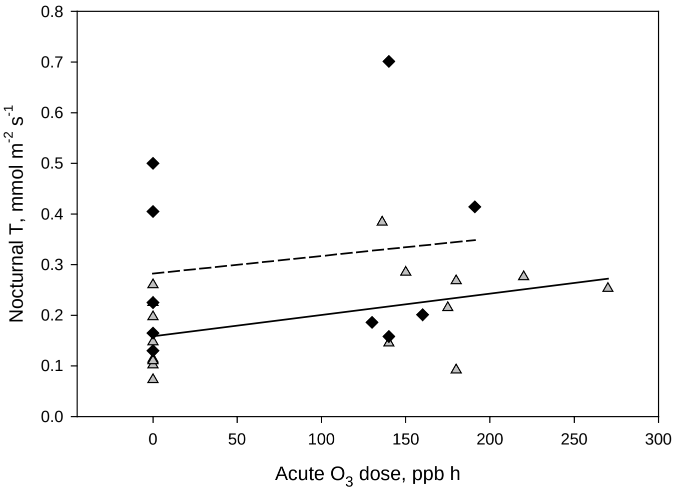

**FIGURE 3.** Nocturnal transpiration (T) of both oaks plotted together for saplings grown in charcoal-filtered air (grey triangles, lower solid line) or grown with chronic $O_3$ exposure (black diamonds, upper dotted line). The effect of short-term acute $O_3$ exposure is suggested by the change from measurements in the control cuvette (on y-axis) vs. the values plotted for the (nonzero) acute $O_3$ dose. The regression lines did not differ significantly.

either species when previously conditioned to chronic daytime $O_3$ exposure. However, in blue oak with no prior $O_3$ exposure, short-term (1 h) acute $O_3$ exposure significantly enhanced nocturnal transpiration. California black oak with no prior $O_3$ exposure was unresponsive to short-term acute $O_3$ exposure.

California black oak had higher daytime gas exchange rates and relatively high nocturnal T (30% that of maximum daytime values) in response to only 1 month of chronic $O_3$ exposure. Chronic $O_3$ exposure over 2 months in blue oak also resulted in increased nocturnal transpiration, but not as much as for California black oak (16% that of maximum daytime values). Although blue oak apparently was more tolerant to chronic $O_3$ exposure, it was more sensitive than California black oak to short-term acute $O_3$ exposure. At night after short-term acute $O_3$ exposure, blue oak had 24% of the maximum daytime T observed. Stimulation of nocturnal transpiration was ephemeral in blue oak; nocturnal transpiration was similar in unexposed and exposed leaves 2 days after acute $O_3$ exposure (data not shown). In *Arbutus unedo*, a Mediterranean tree, a 90-day $O_3$ exposure induced prolonged aberrations in stomatal behavior for up to 10 days[10].

Species of oak are known to emit isoprene and other volatile organic compounds[11] that degrade $O_3$, thus potentially modifying cuvette $O_3$ concentrations. The relative emission of these two oaks is unknown. Because the $O_3$ concentration of the cuvette was measured (average of the $O_3$ concentration entering and exiting the cuvette) and boundary layer was minimized by high fan speeds, the potential for relative differences in isoprene emission between the two species were not expected to play a role in these observations.

Prior exposure of plants to chronic daytime $O_3$ exposure may have preconditioned the antioxidant system[12] such that a short duration, high-$O_3$ exposure was limited in effect. Apoplastic ascorbate is believed to be the first line of defense against $O_3$ uptake, and perhaps such a defense was already in place in this species to minimize the effects of such short-term acute $O_3$ exposure[13]. Because short-term (1 h, acute) $O_3$ exposure elicited little response of foliar gas exchange of California black oak seedlings (without prior O3 exposure), this species may have endogenously more antioxidant defenses relative to blue oak. These responses underscore the need for additional studies of such systems in native species.

Although $O_3$ exposure[3,4] and both $O_3$ and $NO_x$ exposure[5] have been implicated in aberrant, elevated nocturnal transpiration in conifers, the results presented here provide strong experimental evidence for the role of $O_3$ in the foliar loss of water at night in seedlings of two deciduous oaks.

## ACKNOWLEDGMENTS

We thank Dr. Pam Padgett for our use of her open-top chamber facility, and providing the California black oak and blue oak seedlings. The use of trade names is for information only and does not imply commercial endorsement by the U.S. government. A travel grant was awarded to E.P. in 2005 by the short-term mobility program of CNR.
